# High temperatures alter core behaviors of mountain goats (*Oreamnos americanus*), a cold-adapted species

**DOI:** 10.1093/jmammal/gyag064

**Published:** 2026-07-22

**Authors:** Forest P Hayes, Larissa L Bailey, Kenneth R Wilson, Daniel McGrath, Mark Biel, Joel Berger

**Affiliations:** Department of Fish, Wildlife, and Conservation Biology, Colorado State University, 1474 Campus Delivery, Fort Collins, CO 80523, United States; Department of Fish, Wildlife, and Conservation Biology, Colorado State University, 1474 Campus Delivery, Fort Collins, CO 80523, United States; Department of Fish, Wildlife, and Conservation Biology, Colorado State University, 1474 Campus Delivery, Fort Collins, CO 80523, United States; Department of Geosciences, Colorado State University, 1482 Campus Delivery, Fort Collins, CO 80523, United States; Glacier National Park, PO Box 1, West Glacier, MT 59936, United States; Department of Fish, Wildlife, and Conservation Biology, Colorado State University, 1474 Campus Delivery, Fort Collins, CO 80523, United States; Global Programs, Wildlife Conservation Society, 2300 Southern Boulevard, Bronx, NY 10460, United States

**Keywords:** alpine, climate change, glaciers, mountain goat, resource selection, snow

## Abstract

Rapid climate changes are reshaping ecological communities in montane environments. Among those most strongly impacted are cold-adapted organisms that may experience thermal stress at moderate temperatures. The importance of facultative behavioral changes to altered abiotic resources like water, shade, and snow is well documented. However, behavioral patterns of spatial selection may change, particularly for cold-adapted species during summer months. We examine how high summer temperatures affect the behavior of mountain goats (*Oreamnos americanus*), a cold-adapted species with a Holocene distribution closely associated with periglacial environments in western North America. We hypothesized that habitat use by mountain goats during summer months is modulated by high temperatures, leading to increased selection for habitats that offer thermal refuge, including snow. We evaluated resource selection at 2 spatial scales: within the home range and at 4-h movement steps. During the warmest 15% of summer days, at the home range scale, mountain goats increased use of areas with lower solar radiation, higher elevations, locations further from predator escape terrain, and areas closer to human infrastructure. Contrary to expectations, mountain goats exhibited only modest selection for proximity to snow, and selection further decreased at peak temperatures at the home range scale. At the fine scale (4-h steps), individuals selected for relatively lower elevation and increased solar exposure during the warmest periods. These results suggest that avoidance of thermal radiation may be more effective through changes in behavior at the home range scale. Importantly, we document changes in intrinsic behaviors such as seeking safety from predators in steep terrain during the warmest days. As montane environments continue to warm rapidly, understanding changes in resource use during the warmest periods today offers an invaluable lens for understanding challenges for populations tomorrow.

## Teaser

As montane environments warm, cold-adapted species like mountain goats may alter behavior to reduce heat stress. During the warmest periods, we found increased use of areas that are cooler, near humans, and further from escape terrain, highlighting climate-driven changes.

Anthropogenic climate change is indelibly altering ecosystems and presents a key threat to long-term conservation of biodiversity (Lovejoy and Hannah 2019; White et al. 2025). In past epochs, periglacial zones provided refuge for cold-adapted species (Pielou 2008; Rosvold 2016) but as Earth warms, species inevitably die out, shift ranges, or develop adaptations to changing thermal conditions (Parmesan 2006; Rowe et al. 2015; Wiens 2016). In alpine ecosystems, temperatures are warming 2 to 5 times more rapidly than elsewhere, resulting in loss of glacial mass, decreased snow persistence, and changes in seasonal phenology (Choi et al. 2010; Hugonnet et al. 2021; Pörtner et al. 2021). Cumulative thermal alterations have resulted in striking range collapses of several cold-adapted mammals along the southern peripheries of their ranges, including American Pika (*Ochotona princeps*), Yellow-bellied Marmot (*Marmota flaviventris*), and Least Weasel (*Mustela nivalis*; Hayes and Berger 2023).

The species that perhaps best typifies a tight association between distribution and periglacial zones is the Mountain Goat (*Oreamnos americanus*; White et al. 2025). Unlike related and more widespread ungulates, including Bighorn Sheep (*Ovis canadensis*) and Ibex (*Capra* spp.), extant mountain goats have narrow ecological niches in relatively cold and snowy environs (Festa-Bianchet and Côté 2008). Their native range is inextricably tied to the cold Pacific rugged mountain realm in North America coupled with interior montane glaciated systems (Fig. 1). Because they are reliant on cold environments, those found at low latitudes or low elevations are often the first to be impacted by a warming climate (Wiens 2016; Hayes and Berger 2023). Notably, both the Mountain Goat and an associated congeneric (*O. harringtoni*) have become extirpated in warming Holocene climes from northern Mexico, Arizona, and Utah (Rideout and Hoffmann 1975; Hayes and Berger 2023). Further, introduced populations that do exist at lower latitudes are reliant on isolated high-elevation refugia (Hayes and Berger 2024).

**Fig. 1:**
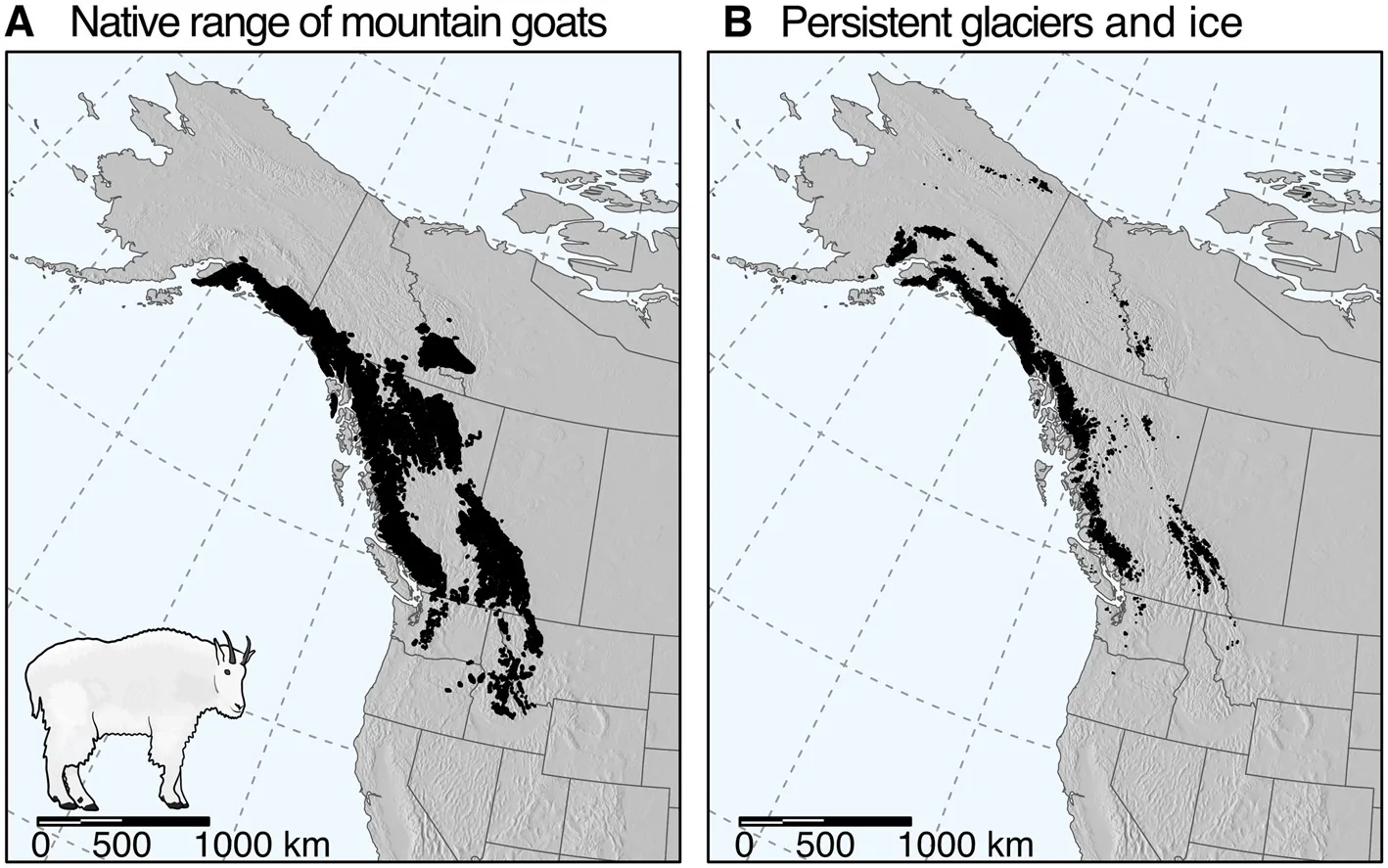
The native distribution of mountain goats (*Oreamnos americanus*) and periglacial zones. (A) The extant range of mountain goats (Mountain Goat Management Team 2010). (B) The distribution of persistent glaciers and ice in western North America (data from North American Atlas).

In response to warming temperatures, animals make both fine-scale alterations in resource use and broad-scale shifts in locality. Such behavioral shifts are observed in many ungulates (Merrill 1991; Dussault et al. 2004; Van Beest et al. 2012; Rigoudy et al. 2025) and diverse taxa, including ice rats, pikas, and marmots (Melcher et al. 1990; Hinze et al. 2006; Smith 2020). Moose (*Alces alces*), a Holarctic species, reduce movement and increase use of shaded areas and standing water for conductive cooling (Renecker and Hudson 1990; Alston et al. 2020; Verzuh et al. 2023). Similarly, fine-scale changes in temporal activity patterns have been observed in Alpine Ibex (*C. ibex*) and Caribou (*Rangifer tarandus*) which exhibit reduced activity during warm periods and increased activity during cooler portions of the day (Aublet et al. 2009; Trondrud et al. 2023). At broader scales, alpine species are shifting to higher elevations (Parmesan 2006), with changes noted for mammals including pika and Crested Porcupine (*Hystrix cristata*; Moritz et al. 2008; Mori et al. 2018). Further, low-latitude populations of some species have been extirpated from warmer reaches in recent times (Chen et al. 2011; Hayes and Berger 2023). Among others, marmots (*Marmota* spp.), Caribou, and Snowshoe Hare (*Lepus americanus*) have all become extirpated from the southern portion of their historical ranges (Racey and Armstrong 2000; Armitage 2013; Wilson et al. 2022), and Caribou are now altering migration pathways and using more mountainous portions of their range due to changing ambient conditions affecting nutritional availability and overwinter survival (Gurarie et al. 2024).

Recent global warming is associated with decreased persistence of snow and greater seasonal variability in mountainous regions (Choi et al. 2010). A multitude of species make use of snow which provides various ecological benefits, including foraging habitat, relief from heat and insects, and water sources (Rosvold 2016). For mountain goats, snowpack persisting into summer may provide both heat relief and a refuge from insects (Sarmento et al. 2019; Hayes and Berger 2023). However, the functional role of ephemeral snow has been sparsely studied and prior studies have only evaluated correlative evidence. Less clear for many species are the physiological and ecological costs associated with a lack of snow. In contrast, the direct negative effects of deep snow are comparatively well established and include increased energetic expenditure for locomotion, increased predation risk, and death by avalanche (Parker et al. 1984; Huggard 1993; White et al. 2011, 2024). Despite the putative benefits of snow, we have limited understanding of the role of persistent snow in modulating habitat suitability and spatial resource selection.

Among species of montane environments, changing relationships between summer temperatures and habitat use reflect important behavioral choices (Teitelbaum et al. 2021). Selection for shade and altered patterns of foraging during cooler periods of the day are prime examples and may take place at multiple different spatial and temporal scales (Aublet et al. 2009; Mason et al. 2017; Alston et al. 2020). However, use of summer snow patches and possible tradeoffs between thermal constraints and predation risk remain understudied—and yet are critical to understanding potential adaptive responses to future challenges (Veldhuis et al. 2019).

Under continued climate trends, species outcomes will be influenced by their capacity to make adaptive changes in habitat use (Thomas‐Kuzilik et al. 2025). Given that mountain goats have limited ability to shift home ranges upward in elevation and face barriers to long-distance movement, plasticity in behavior at the home-range scale and smaller will be especially important (Harris et al. 2022; Michaud et al. 2024). We investigate how populations near the southern terminus of their native range respond to periods of high daily temperatures. We hypothesized that habitat use by mountain goats during summer months is modulated by temperature, especially during warmer periods, and results in increased selection for habitats that facilitate heat avoidance. If so, we expected to see individuals exhibit greater selection for resources associated with heat avoidance, such as higher elevations, shaded aspects, and persistent snow.

To test our hypothesis, we contrasted habitat use of mountain goats during the warmest days of the year against cooler periods to understand behavioral shifts under increasingly warm conditions. We used daily remote-sensed snow presence data across a 10-year period to evaluate the effects of changing snow availability on resource use. By comparing resource selection during these periods, we aim to better understand the consequences of warming temperatures for a species that is reliant on cold environments (Hall and Fagre 2003; White et al. 2018).

## Methods

### Sample area

Glacier National Park (48.8° N, −113.8°W) is a large (4,100 km^2^) protected area characterized by rugged high-elevation mountains dominated by alpine tundra and glacial lakes. Lower elevations are typified by coniferous forests and intermittent open meadows. Extant predators of mountain goats include grizzly bears (*Ursus arctos*), black bears (*Ursus americanus*), mountain lions (*Puma concolor*), wolves (*Canis lupus*), and coyotes (*Canis latrans*; https://doi.org/10.1002/ecm.1509, Sarmento and Berger 2017). Mountain goats are native to the region with an estimated population size of 2,000 to 3,000 in Montana (Festa-Bianchet and Côté 2008).

### Spatial data and mountain goats

We coupled satellite imagery with GPS locations from collared mountain goats to generate a novel assessment of the changes in resource use during periods of peak temperatures while accounting for snow availability on the date of location use (Fig. 2). We captured mountain goats (*n *= 30) in Glacier National Park, United States using either Clover traps or ground darting, and fitted satellite GPS collars during 2013 to 2014 (Sarmento et al. 2019) and 2018 to 2020 with 2- or 4-h fix rates (Supplementary Data SD1). We captured, handled, and observed mountain goats in accordance with applicable guidelines from the American Society of Mammologists (Sikes et al. 2016) and with approved institutional animal care and use committee protocol (Glacier National Park IMR_GLAC_Biel_MtnGoat_2018.A3).

**Fig. 2:**
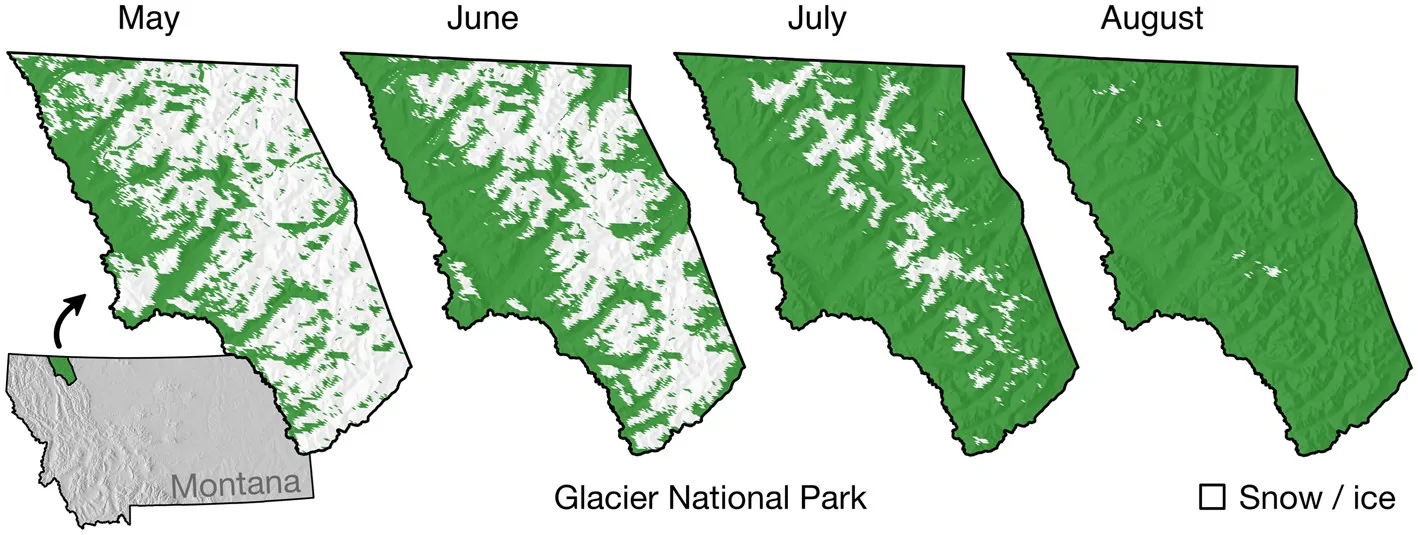
Seasonal snow and ice phenology in Glacier National Park, MT. Panels illustrate the monthly reduction in snow extent during May to August for a single study year (2022). Data are from the MODIS Terra Daily NDSI product.

Monitoring yielded 88 animal-years of data (mean 2.93, SD 0.83 years/animal, range 1 to 5 years). We recorded 67,215 GPS locations from 8 individuals between 2013 and 2016 and 98,497 GPS locations from 22 individuals between 2018 and 2022. For data collected between 2018 and 2022, which included horizontal location error, we censored 10,842 points with error exceeding the resolution of our spatial dataset (i.e., >30 m). We then created a subset of locations representing the summer period when snow is receding (Fig. 2; from 15 May to 31 August), resulting in a total of 47,950 observed locations.

We used elevation data with a 30 m spatial resolution from the USGS National Elevation Dataset (USGS 2005) and derived slope and aspect from the elevation using the R package “raster” (Hijmans 2021). We classified slopes (≥33°) that offer refuge from predators as escape terrain (Gross et al. 2002), and generated a distance-to-escape layer using the GRASS GIS (GRASS Development Team 2019). Based on slope and aspect, we calculated daily solar radiation per unit area for each day $(\mathrm{watt}-\mathrm{hour}\times m^{2})\;$ for the entire study region. First, we calculated visible horizon from the center of each 30 m grid cell in 5° intervals using the digital elevation model. We used fixed baseline parameters for surface albedo (i.e., ground reflectance, 0.22) atmospheric transmissivity (clear sky conditions; Marshall and Miller 2020). We then calculated daily solar radiation for each grid cell with the “r.sun” function in GRASS GIS and used this as a representative measure of thermal exposure across our study area.

We used distance to roads and trails to incorporate a measure of potential anthropogenic presence as a possible modifier of mountain goat behavior. We acquired vector data from Open Street Map (www.openstreetmap.org) and converted them to 30 m resolution raster images. We then calculated least distance-to-feature for the center location of each grid cell using GRASS GIS.

### Assessing snow cover

We measured the last day that snow was present during summer using the MODIS satellite NDSI data product (Hall et al. 2006). While the spatial resolution of these data is relatively low (500 m), the high temporal sampling resolution offering daily coverage is critical to capture the rapid melt of remnant snow during summer months (Fig. 2). Importantly, for each year, we identified snow as pixels with ≥10% snow cover for at least 2 weeks. We additionally masked (i.e., excluded) areas predominantly covered by water during summer months to avoid misclassification as snow. We identified the last day of the summer for each year where snow cover was ≥3%. All MODIS data were processed using Google Earth Engine. We determined snow to be present and accessible by mountain goats if the date of use was on or before the last day of the year with recorded snow cover.

### Identifying periods of peak temperature

We identified periods with the warmest temperatures by assessing the warmest 15% of days per year during the summer study period. We used daily mean air temperature from a centrally located weather station (SNOTEL #482; 48.8° N, −113.85° W; 6,300 ft; www.nrcs.usda.gov). We chose mean daily air temperature as it encapsulates warmth across an entire 24 h day; however, days with the top 15% minimum and maximum temperatures were also similar (Supplementary Data SD2). We created subsets of used and available location data (described below) based on the warmest days (top 15% of days) and cooler days (all other days) during summer (15 May to 31 August).

### Resource selection analysis

We assessed availability of resources at 2 spatial scales: within the home range of an individual animal and within a 4-h movement step (a distance drawn from the observed 4-h movement distribution) from each used location. We estimated the annual summer home range of each individual using a 95% minimum convex polygon with the R package “sp” (Supplementary Data SD3). This method provides a simple home range estimate and has been shown to produce unbiased covariate estimates when applied (Fieberg and Börger 2012).

To identify resource selection at a finer scale, we used a step-selection process (Thurfjell et al. 2014). First, we created 4-h steps (i.e., distance and direction of movement between recorded locations) using a minimum of 3 consecutive points, and a tolerance of 30 min using the R package “amt” (Signer et al. 2019). Steps had a median distance of 658 m with a right-skewed distribution typical of frequent short-distance movements and infrequent long-distance movements (Supplementary Data SD4). We assessed resources based on availability at the date and time of observed locations as some spatial data were temporally variable. For each spatial scale, we extracted values for used locations based on the geographic coordinates and time stamp of each location. We generated 10 random available locations for each recorded used location with availability for our 2 spatial scales determined by individual home range and movement based on a random step length and turning angle drawn from the distribution of observed values (Northrup et al. 2013; Street et al. 2016). We evaluated resource selection by comparing environmental attributes at observed locations to those at random locations, defining selection as disproportionate use of a resource relative to its availability at a given spatial scale.

We modeled distance-based resources as distance-to-feature and, to facilitate interpretation, we evaluated selection as proximity-to-feature (i.e., the inverse of distance-to-feature) such that positive selection indicates increased proximity to the resource. For proximity-to-feature, we transformed observed distance-to values using $\mathrm{lo}g_{10}(x+1)$ to meet assumptions of normality and facilitate model fit. For proximity to snow, we restricted the effect to distances less than 2 km to reflect the low probability of direct resource use based on observed movement distances between GPS fixes (Supplementary Data SD4). We centered and scaled all covariates by subtracting the mean and dividing by standard deviation. Pairwise correlations between all covariates were low (|r|<0.4), indicating little collinearity. We used a Bayesian hierarchical random effect resource selection model (Thomas et al. 2006) to evaluate summer (i.e., 15 May to 31 August) selection by mountain goats at 2 spatial scales and account for individual variation. We used a uniform distribution for the population-level hyperparameter (α) for each covariate (x):


$$\alpha_{x}\;=\;U(\min=0,\max=25)$$

with a population-level variance:


$$\tau_{x}=N(\mu =0,\sigma =0.001).$$

We treated individual (*i*) as a random effect with the prior distribution for each covariate ($\beta_{x}$) informed by population-level distributions:


$$\beta_{x,i}=N(\mu =\alpha_{x},\sigma =\tau_{x})$$

We modeled the relative probability (p) of selection for resources by each individual as


$$\mathrm{logit}(p_{i,j,k})=\beta_{0}+\beta_{1,i,j,k}*x_{1,i,j,k}+\;\ldots \;+\beta_{n,i,j,k}*x_{n,i,j,k}$$

where $p_{i,j,k}$ is the relative probability of selection by individual *i* at observation j, scale k, $\beta_{0}$ is the intercept, and $\beta_{1}\ldots \beta_{n}$ are coefficients estimated for covariates $x_{1}\ldots x_{n}$. We modeled the likelihood of use by individual i at observation j and scale k (i.e., $\mathrm{pre}s_{i,j,k}$) as a Bernoulli random variable:


$$\mathrm{pre}s_{i,j,k}=\mathrm{Bernoulli}(p_{i,j,k}).$$

This model structure allows for sharing of information between individual-level effects while accounting for individual variation and differences in sample sizes (Thomas et al. 2006). For each spatial scale, we determined population-level effects by taking the mean of all individual-level effects (i.e., $\mu_{\beta x}$). We evaluated a single global model which included all covariates of interest and determined the effect based on the magnitude of beta estimates and the 95% credible interval. We ran models for 5,000 iterations and 2,500 iterations of burn in at which point all model estimates had reached convergence (R̂ < 1.01; Gelman and Hill 2007).

## Results

### Selection within home range

Escape terrain, elevation, human presence, and solar radiation were key aspects of space use by mountain goats within their home range (Fig. 3; Supplementary Data SD5). Anthropogenic features were influential, with positive selection for proximity to both roads and trails. Moreover, snow had the smallest effect of any covariate at the home range scale (Fig. 3). Habitat use was positively associated with topography characterized by increased solar radiation, although, like proximity to snow, the relative effect size was small (Fig. 3).

**Fig. 3:**
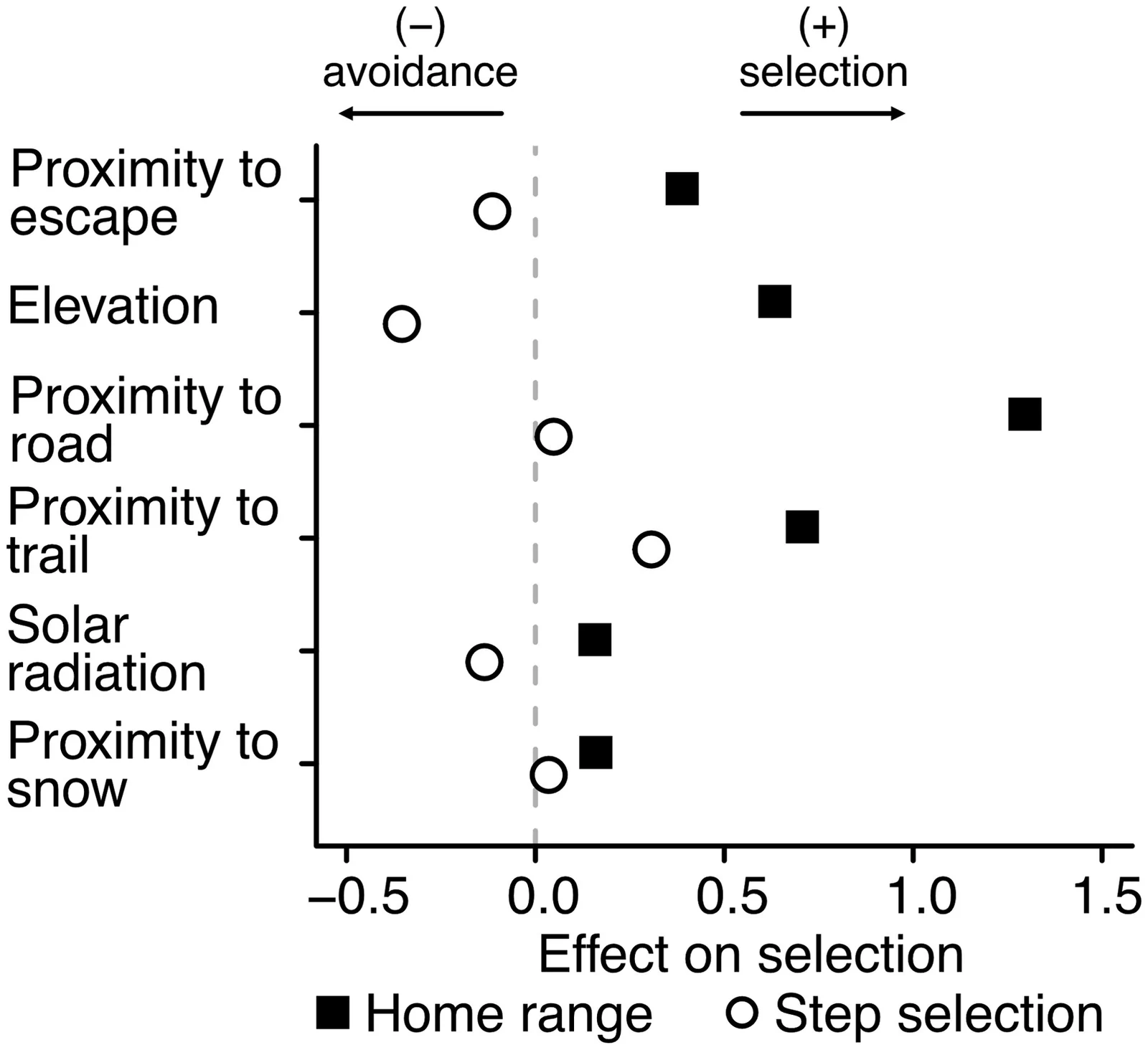
Population-level effects of habitat covariates (*y*-axis) on resource selection by mountain goats (*Oreamnos americanus*; *n *= 30) in Glacier National Park from 15 May to 31 August during 9 years within 2013 to 2022. The *x*-axis shows covariate estimates representing the relative effect on selection with greater absolute values indicating increased use of a resource relative to its availability. Points represent mean estimates of selection for each covariate, with square points indicating resource availability measured at the home range scale and circles showing resource availability based on a 4-h step selection process. All covariate estimates had statistically significant effects with credible intervals not including zero. Credible intervals for all variables are within ± 0.03 of mean estimates and are reported as numeric values in Supplementary Data SD5.

On average, the warmest 15% of summer days were 7.44 °C (SD 0.66) warmer than cooler days (Table 1). On the warmest days, mountain goats showed reduced preference for escape terrain, solar radiation, and snow; instead, they selected for higher elevations and areas with greater proximity to anthropogenic features (Fig. 4; Supplementary Data SD6). However, the broader credible intervals for selection during the warmest days may be a consequence of reduced data availability rather than increased variance in estimated individual effects.

**Fig. 4:**
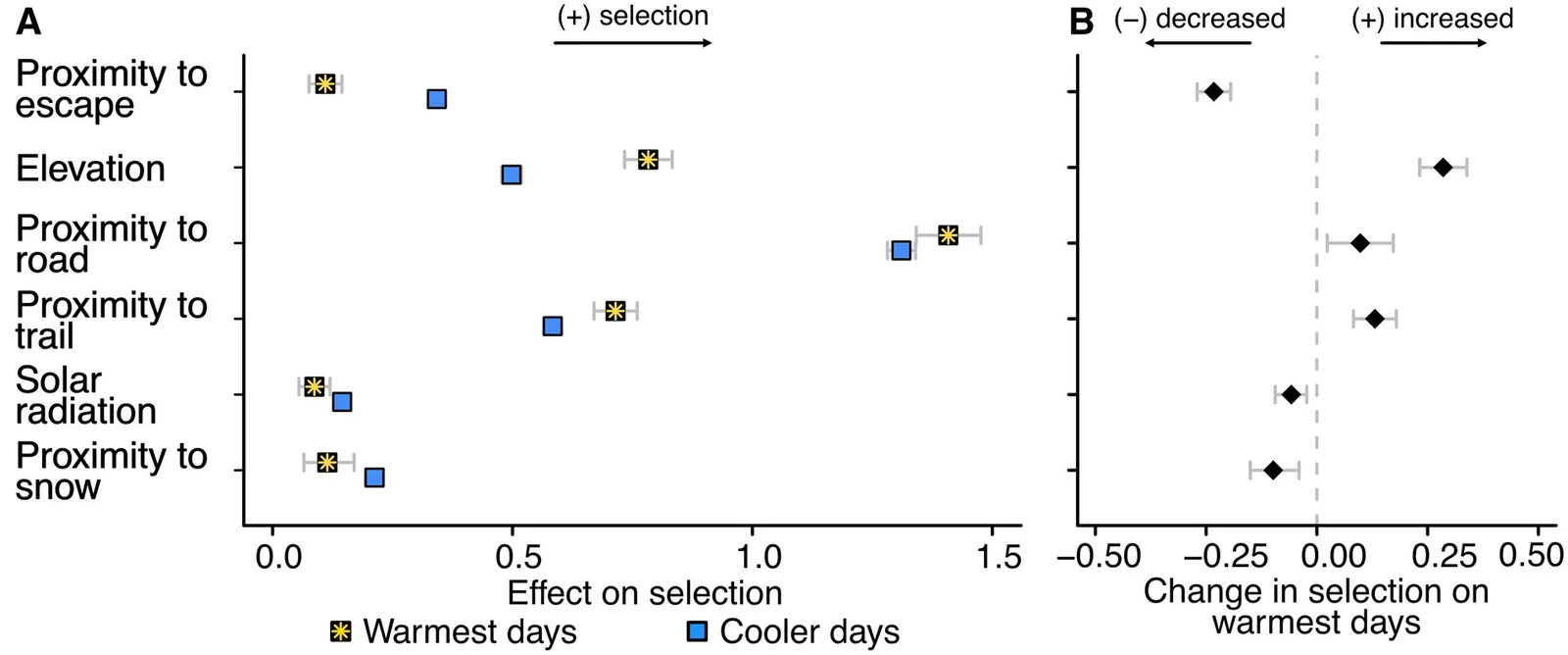
(A) Effects of habitat covariates (*y*-axis) on resource selection by mountain goats (*Oreamnos americanus*; *n *= 30) within the home range during the warmest (top 15% of days) and cooler (all other) days during summer (15 May to 31 August) during 9 years within 2013 to 2022 in Glacier National Park. (B) Change in magnitude and direction of change in selection during the warmest days relative to selection on cooler days. The *x*-axis represents relative effect on selection with greater absolute values indicating increased use of a resource relative to its availability. Points represent mean estimates of selection and whiskers represent the 95% credible intervals. Covariate estimates with credible intervals not overlapping zero and nonoverlapping estimates between periods are considered statistically significant. Points without whiskers have credible ranges of the mean value ± 0.02 and are provided with numeric results in Supplementary Data SD6.

**Table 1:** Yearly mean ± SD of mean daily temperature in degrees Celsius for the warmest 15% of days (*n *= 16/year), calculated annually, and cooler days (*n *= 93/year) during 15 May to 31 August in Glacier National Park.

	Year								
Category	2013	2014	2015	2016	2018	2019	2020	2021	2022
**Cooler days**	10.07 ± 3.74	9.13 ± 3.44	9.90 ± 3.76	8.29 ± 3.57	9.91 ± 3.22	9.20 ± 3.24	9.30 ± 3.55	10.89 ± 4.95	9.91 ± 4.69
**Warmest days**	16.93 ± 1.24	16.01 ± 0.79	18.14 ± 1.47	15.13 ± 0.54	17.32 ± 1.36	15.80 ± 1.15	17.40 ± 1.16	19.04 ± 1.08	17.78 ± 0.69

Selection for topographic variables (escape terrain and elevation) had the greatest change between hot and cool days (Fig. 4B). Mountain goats demonstrated a substantial increase in selection for higher elevations during hot days and concurrently exhibited a substantial decrease in selection for proximity to escape terrain. Individuals also showed increased use of areas in proximity to roads and trails, although the relative change in selection for these covariates was smaller.

Selection for higher levels of solar radiation showed a modest decrease during the warmest days (Fig. 4). While mountain goats still occupied terrain with higher solar exposure relative to availability, the strength of selection was reduced in comparison to cool days. This result suggests that increased thermal stress during high ambient temperatures may encourage use of slopes with less solar exposure. Mountain goats also exhibited reduced selection for proximity to snow during hot days, suggesting that use of areas proximate to snow do not confer as strong of thermoregulatory benefits as might have been expected.

### Fine-scale selection

We found that proximity to snow had only a minimal effect on fine-scale space use (Fig. 3). Additionally, individuals exhibited reduced selection for high-elevation areas and areas near escape terrain in comparison to the broader scale. In another noteworthy contrast, mountain goats exhibited avoidance of solar radiation at the fine scale, indicating that smaller movements may be used to reduce thermal exposure.

There were substantial differences in fine-scale resource selection between the warmest and cooler days (Fig. 5; Supplementary Data SD7). Notably, during the warmest days, changes in selection for proximity to escape terrain, elevation, and solar radiation were in the opposite direction relative to results at the home range scale. This contrast indicates greater importance of these resources at larger spatial scales and shows how changes in the availability of nearby habitat (i.e., because of movement within their home range) influence selection. Mountain goats also used habitats that were further away from areas identified as snow. The consistency of results at differing spatial scales provides evidence that use of snow habitats is unlikely to increase under warming summer temperatures.

**Fig. 5:**
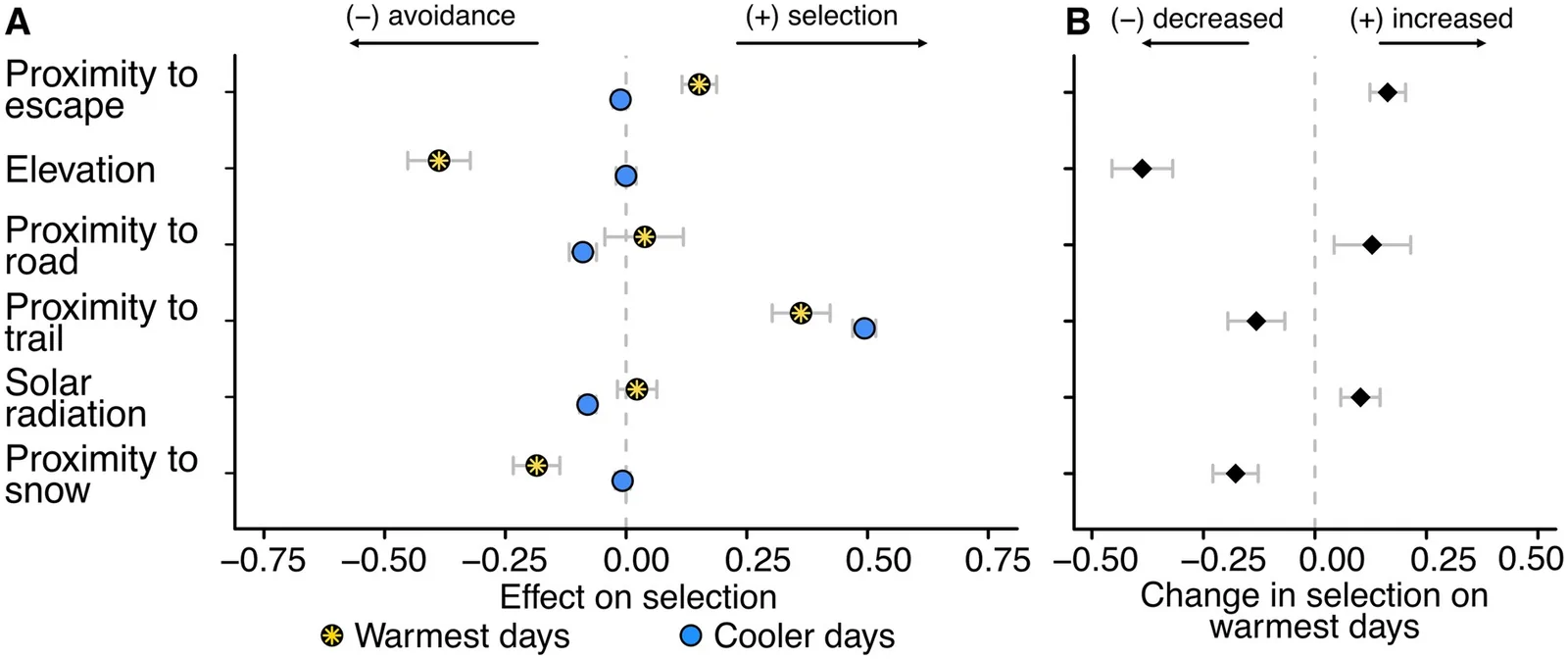
(A) Effects of habitat covariates (*y*-axis) on resource selection by mountain goats (*Oreamnos americanus*; *n *= 30) with availability based on 4-h steps during the warmest (top 15% of days) and cooler (all other) days during summer (15 May to 31 August) during 9 years within 2013 to 2022 in Glacier National Park. (B) Change in magnitude and direction of change in selection during the warmest days relative to selection on cooler days. The *x*-axis represents relative effect on selection with greater absolute values indicating increased use of a resource relative to its availability. Points represent mean estimates of selection and whiskers represent the 95% credible intervals. Covariate estimates with credible intervals not overlapping zero and nonoverlapping estimates between periods are considered statistically significant. Points without whiskers have credible ranges of the mean value ± 0.02 and are provided with numeric results in Supplementary Data SD7.

## Discussion

The question confronting our understanding of animals under changing climate conditions is no longer whether or not they exhibit behavioral flexibility. Instead, it is the extent to which species or populations are capable of change under increasingly extreme conditions. Already, warming temperatures have resulted in broad shifts in distribution and behavior of numerous montane species (McCain et al. 2021; Thomas‐Kuzilik et al. 2025). Our attempt to address these issues for mountain goats—a species reliant on peri-glacial environs—has shown that animals make noteworthy changes in space use during the hottest days of the summer.

The relative ability (or inability) of endotherms to dissipate heat has been put forth as a potential factor limiting fecundity (Speakman and Król 2010), especially important for mountain goats, which already have low reproductive rates. Mountain goats may be increasingly predisposed to thermal stress due to phenological mismatches such as mistimed molting (Festa-Bianchet and Côté 2008; White et al. 2025). With climate change leading to rapid warming of alpine regions (Pörtner et al. 2021), behavioral changes in space use represent an important mechanism by which cold-adapted species may mitigate thermal challenges (Thomas‐Kuzilik et al. 2025).

The importance of persistent snow, ice, and periglacial zones for cold-adapted species has been insufficiently established despite strong species associations with these environments (Fig. 1). Overall, our assessment of space use by mountain goats was in close agreement with previous literature (e.g., Gross et al. 2002; Poole et al. 2009; Richard and Côté 2016). Notably, however, we found that selection for snow is not increasing under hotter conditions as would have been expected if snow-dominated habitats were providing a refuge from increased temperatures. This finding contrasts with prior research in this region which indicated that mountain goats preferentially select for areas with annually persistent snow and ice (Sarmento et al. 2019). This difference in results likely stems from methods used to estimate snow availability. Namely, annually persistent snow and ice are tightly related to topography, while ephemeral snow availability declines throughout the summer (Fig. 2). Thus, prior results may be, in part, explained by a greater correlation between annually persistent snow and ice and steep aspects at high elevations.

Avoidance of deep snow occurs in large-bodied, cold-adapted species due to increased locomotion costs (Parker et al. 1984; Dailey and Hobbs 1989; White and Gregovich 2017). However, these effects are reduced during summer months when snow is less deep and coverage is patchy. It is possible that mountain goats are still selecting for snow but at spatial and/or temporal resolutions that are not captured by our empirical data. That said, observations of individual physiological responses suggest that mountain goats likely derive little thermal benefit from bedding on snow (Hayes and Berger 2023). Thus, our findings here—that mountain goats do not select for areas characterized by snow during times of peak temperatures—are less surprising and further suggest that a reduction of persistent snow and ice alone is unlikely to create increased thermal challenges for mountain goats. A distinct possibility is that mountain goats are using alternative methods for avoiding thermal stress—such as shifting diel activity patterns or using shaded microsites—which were unable to be captured by our analysis.

We found support for high-temperature-mediated changes in resource selection, including reduced use of areas with high solar exposure and increased use of higher elevation locations (Figs 4 and 5). Such elevational shifts are well supported across numerous taxa and may provide thermal relief due to reduced air temperatures at higher elevations (Rolland 2003; Parmesan 2006; Moritz et al. 2008). Reduced selection for thermal exposure at the home range scale suggests that observed changes are, at least in part, a result of increased ambient temperatures. Although predation risk was not directly measured in this study, there was a notable concurrent increase in use of areas associated with higher risk of disturbance or mortality (i.e., further from escape terrain and closer to humans; Festa-Bianchet and Côté 2008). Such a tradeoff between thermal stress and predation exposure is likely for large herbivores but has not been tested extensively (Veldhuis et al. 2019). Increasing use of areas farther from escape terrain during the warmest periods suggests that temperature-induced shifts in resource use may expose mountain goats to higher predation risk. However, we also observed a smaller corresponding increase in selection for escape terrain at the fine scale, which may partially offset these effects. As the ecological benefits of these covariates are well established for mountain goats, this pattern suggests that these critical resources are of greater importance at broader scales, such as within home range.

The relative costs and benefits of increased use of areas close to anthropogenic activity are less clear. On the positive side, mountain goats gain increased access to anthropogenic sources of salt including human urine, and the presence of people offers an apparent human shield from predators (Singer 1975; Berger 2007; Sarmento and Berger 2017). Recent research also indicates that mountain goats reduce use of locations associated with humans when humans are excluded from the landscape (e.g., due to recreation closures; Gaynor et al. 2025). However, a growing body of evidence also suggests that anthropogenic disturbance may have substantial energetic costs (Neumann et al. 2011). Although we are unable to disentangle these effects in our work, the fact that use of areas proximate to anthropogenic activity increased during the warmest days of the year may suggest a complicated tradeoff between predation risk and thermal costs that warrants additional attention.

Overall, our results point to temperature-modulated resource selection and suggest that increased selection for snowy locales is unlikely, even as glaciers diminish and climes warm. Additionally, changes in behavior related to avoidance of thermal exposure can lead to increased use of high-risk habitats, heightening the chances of predation or energetically costly disturbance. These behavioral shifts are especially noteworthy because mean summer temperatures in Glacier National Park are anticipated to reach 17.30 °C—levels similar to the top 15% of days in our study—by the end of the century (White et al. 2025). As warmer temperatures reduce the availability of cooler habitats, species may exhibit shifts in resource use and tradeoffs between thermal environment and safety. Under future climate conditions, the indirect effects of changing behavior, such as exposure to increased predation risk, are a key concern as elevational and geographic shifts in resource use must, we assume, be necessary for survival. As populations at the marginal edge of distributions will be most strongly impacted, evaluating responses to the warmest experienced conditions, as we have elected to do, provides insights about future population responses and challenges to long-term persistence.

## Supplementary Material

gyag064_Supplementary_Data

## Supplementary data

**Supplementary Data SD1**. Numeric summary of captured mountain goats by year and sex.

**Supplementary Data SD2**. Comparison of metrics identifying the hottest summer days using daily maximum, minimum, and average temperatures from SNOTEL #482.

**Supplementary Data SD3**. Annual Mountain Goat home range size estimates from 2013 to 2022.

**Supplementary Data SD4**. Histogram of 4-h step lengths for mountain goats during summer months.

**Supplementary Data SD5**. Numeric summary of population-level covariates effects on resource selection by mountain goats.

**Supplementary Data SD6**. Numeric summary of contrasts in resource selection within home range by mountain goats during the warmest and cooler summer days.

**Supplementary Data SD7**. Numeric summary of contrasts in resource selection based on 4-h movement steps by mountain goats during the warmest and cooler summer days.
