# Supplementary material for: High temperatures alter core behaviors of mountain goats (Oreamnos americanus), a cold-adapted species
Source: J Mammal. 2026 Jul 22;107(5):1103–11. doi: 10.1093/jmammal/gyag064 (PMC13722706; doi:10.1093/jmammal/gyag064)
Supplement: Supplementary file 1 [file gyag064_supplementary_data.zip › Supplementary Data SD1.pdf]

Supplementary Data SD1. Summary of initial year of capture and collar deployment for collared mountain goats (*Oreamnos americanus*) in Glacier National Park by year and sex during 2013–2020.

Year	Sex	Number	Collar make	Collar model	GPS fix rate
2013	F	2	Lotek[1]	Iridium Track 2D	2-hour
	M	-			
2014	F	3			
	M	3			
2018	F	7	Telonics[2]	TGW-3477-4	4-hour
	M	-			
2019	F	3			
	M	-			
2020	F	7			
	M	5			
**TOTAL**		30			

[1]Lotek Wireless Inc., Seattle, WA, USA
[2]Telonics, Inc., Mesa, AZ, USA
