# Supplementary material for: High temperatures alter core behaviors of mountain goats (Oreamnos americanus), a cold-adapted species
Source: J Mammal. 2026 Jul 22;107(5):1103–11. doi: 10.1093/jmammal/gyag064 (PMC13722706; doi:10.1093/jmammal/gyag064)
Supplement: Supplementary file 1 [file gyag064_supplementary_data.zip › Supplementary Data SD2.pdf]

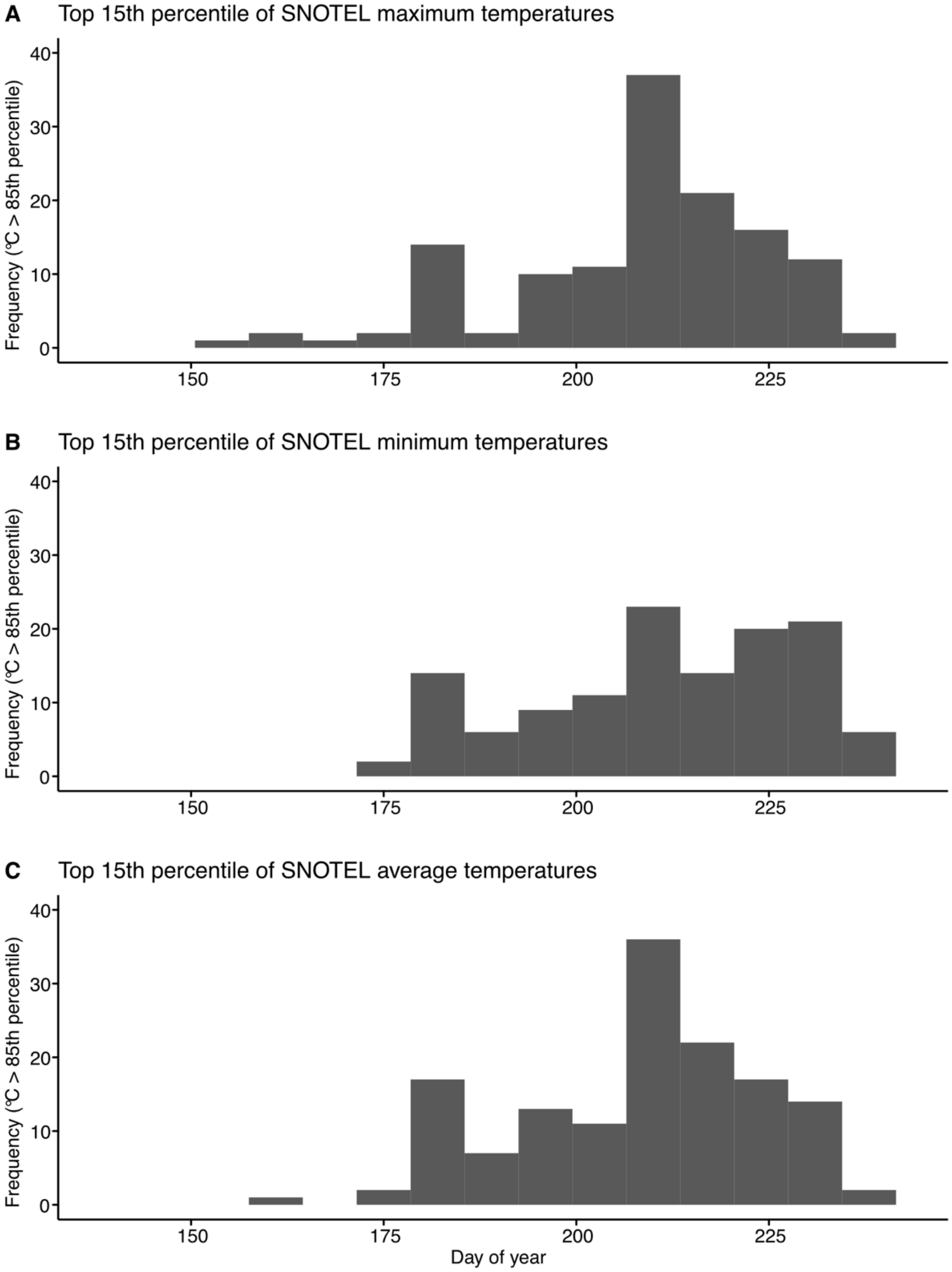

Supplementary Data SD2. Comparison of metrics that reflect the warmest periods of year (top 85th percentile). Data are from (SNOTEL #482; 48.8° N, -113.85° W). Counts represent the frequency of days during each 7-day period during 2013-2016 and 2018-2022 which were identified as being in the top 15th percentile. (A) days identified using daily maximum temperature. (B) days identified using daily minimum temperatures. (C) days identified using daily average temperatures.
