# Supplementary material for: High temperatures alter core behaviors of mountain goats (Oreamnos americanus), a cold-adapted species
Source: J Mammal. 2026 Jul 22;107(5):1103–11. doi: 10.1093/jmammal/gyag064 (PMC13722706; doi:10.1093/jmammal/gyag064)
Supplement: Supplementary file 1 [file gyag064_supplementary_data.zip › Supplementary Data SD3.pdf]

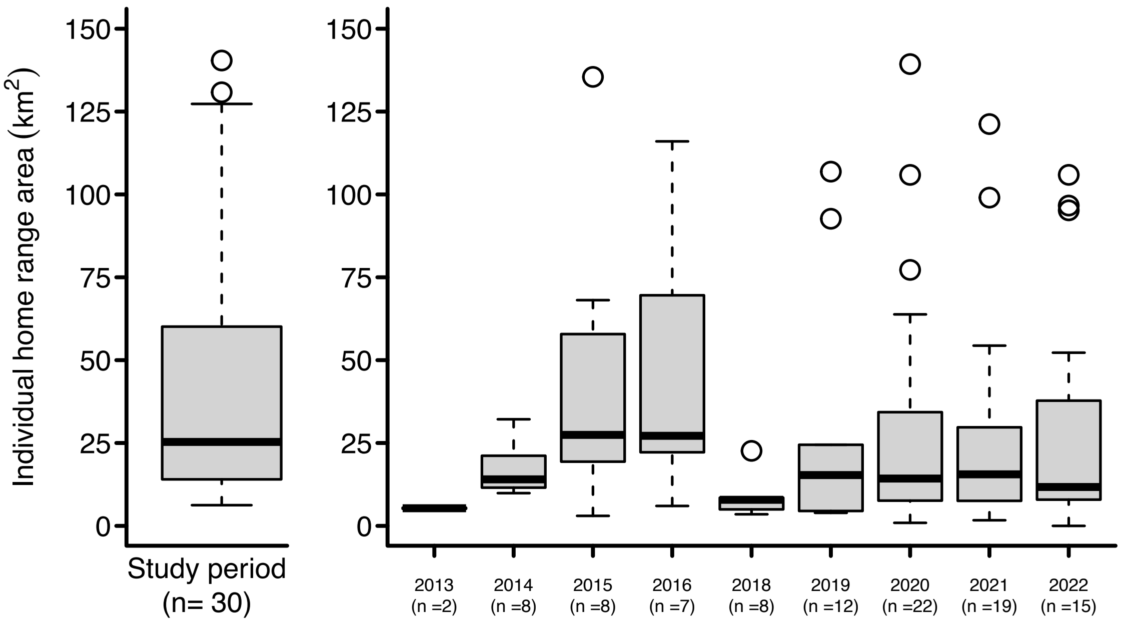

Supplementary Data SD3. Individual Mountain Goat home range size estimates during 2013–2022 in Glacier National Park (left) and individual annual home range size estimates (right). The median value is indicated by a bold horizontal line. Boxes span the interquartile range (25th to 75th percentile), with whiskers extending to the furthest data point within 1.5 times the lower and upper quartiles. Datapoints outside the range of whiskers are marked are outliers and marked with circles. Sample sizes represent the total number of unique individuals observed during the study period and the number of unique individuals observed during a given year, respectively.
