# Supplementary material for: High temperatures alter core behaviors of mountain goats (Oreamnos americanus), a cold-adapted species
Source: J Mammal. 2026 Jul 22;107(5):1103–11. doi: 10.1093/jmammal/gyag064 (PMC13722706; doi:10.1093/jmammal/gyag064)
Supplement: Supplementary file 1 [file gyag064_supplementary_data.zip › Supplementary Data SD4.pdf]

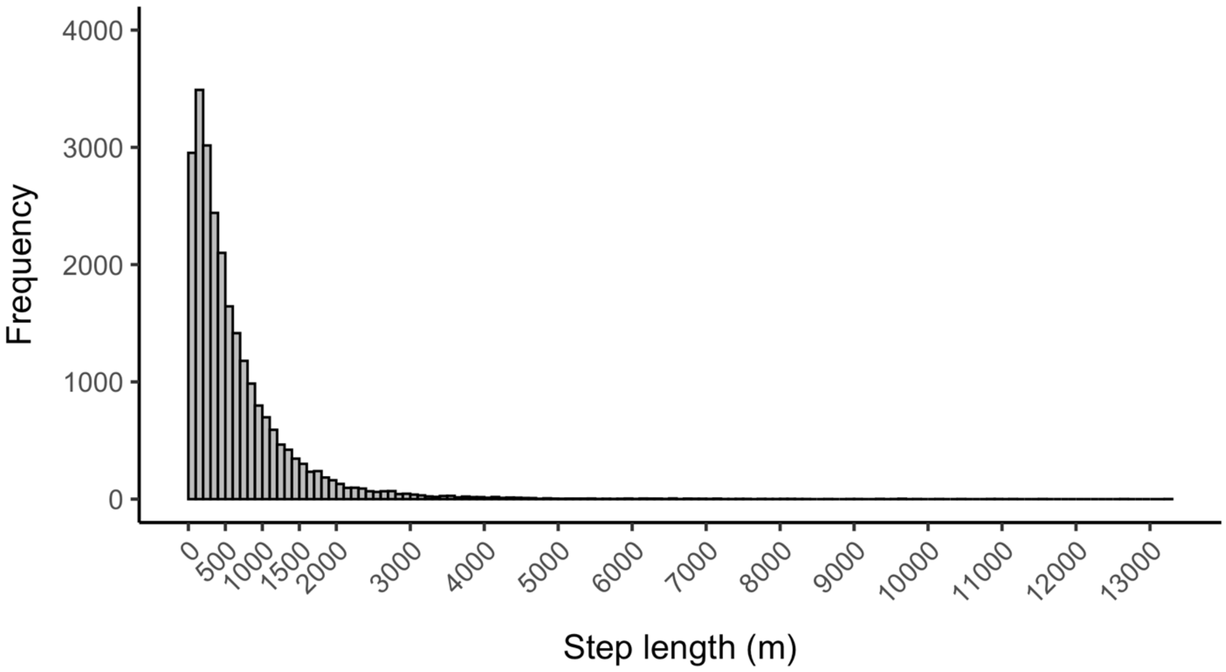

Supplementary Data SD4. Histogram of observed 4-hour step lengths for mountain goats in Glacier National Park, MT during summer months (mid-May to end of August), 2013–2022.
