# Supplementary material for: High temperatures alter core behaviors of mountain goats (Oreamnos americanus), a cold-adapted species
Source: J Mammal. 2026 Jul 22;107(5):1103–11. doi: 10.1093/jmammal/gyag064 (PMC13722706; doi:10.1093/jmammal/gyag064)
Supplement: Supplementary file 1 [file gyag064_supplementary_data.zip › Supplementary Data SD5.pdf]

Supplementary Data SD5. Summary of modeled population-level effects of covariates on resource selection by mountan goats in Glacier National Park during 15 May to 31 August during 2013–2022.

Scale	Variable	Lower CI	Mean	Upper CI
Home range	Elevation	0.62	0.63	0.65
	Proximity to escape	0.37	0.39	0.40
	Proximity to road	1.27	1.30	1.32
	Proximity to trail	0.69	0.71	0.72
	Solar radiation	0.14	0.16	0.17
	Proximity to snow	0.14	0.16	0.18
Step selection	Elevation	-0.38	-0.35	-0.33
	Proximity to escape	-0.13	-0.11	-0.10
	Proximity to road	0.02	0.05	0.07
	Proximity to trail	0.29	0.31	0.33
	Solar radiation	-0.15	-0.13	-0.12
	Proximity to snow	0.02	0.04	0.05
