# Supplementary material for: High temperatures alter core behaviors of mountain goats (Oreamnos americanus), a cold-adapted species
Source: J Mammal. 2026 Jul 22;107(5):1103–11. doi: 10.1093/jmammal/gyag064 (PMC13722706; doi:10.1093/jmammal/gyag064)
Supplement: Supplementary file 1 [file gyag064_supplementary_data.zip › Supplementary Data SD6.pdf]

Supplementary Data SD6. Summary of contrasts between resource selection within home range during the warmest (> 85th percentile) and cooler (all other) days by mountan goats in Glacier National Park during 15 May to 31 August across a decade (2013–2022). Under temperature, "difference" represents the change in selection from cooler to warmest days.

Temperature	Variable	Lower CI	Mean	Upper CI
Cooler	Proximity to escape	0.33	0.34	0.36
Cooler	Elevation	0.48	0.50	0.52
Cooler	Proximity to road	1.28	1.31	1.34
Cooler	Proximity to trail	0.57	0.58	0.60
Cooler	Solar radiation	0.13	0.14	0.16
Cooler	Proximity to snow	0.19	0.21	0.23
Warmest	Proximity to escape	0.08	0.11	0.14
Warmest	Elevation	0.73	0.78	0.83
Warmest	Proximity to road	1.34	1.41	1.48
Warmest	Proximity to trail	0.67	0.71	0.76
Warmest	Solar radiation	0.05	0.09	0.12
Warmest	Proximity to snow	0.07	0.11	0.17
Difference	Proximity to escape	-0.27	-0.23	-0.19
Difference	Elevation	0.23	0.29	0.34
Difference	Proximity to road	0.02	0.10	0.17
Difference	Proximity to trail	0.08	0.13	0.18
Difference	Solar radiation	-0.09	-0.06	-0.02
Difference	Proximity to snow	-0.15	-0.10	-0.04
