# Supplementary material for: High temperatures alter core behaviors of mountain goats (Oreamnos americanus), a cold-adapted species
Source: J Mammal. 2026 Jul 22;107(5):1103–11. doi: 10.1093/jmammal/gyag064 (PMC13722706; doi:10.1093/jmammal/gyag064)
Supplement: Supplementary file 1 [file gyag064_supplementary_data.zip › Supplementary Data SD7.pdf]

Supplementary Data SD7. Summary of contrasts between resource selection based on 4-hour movement steps during the warmest (> 85th percentile) and cooler (all other) days by mountan goats in Glacier National Park during 15 May to 31 August across a decade (2013–2022). Under temperature, "difference" represents the change in selection from cooler to warmest days.

Temperature	Variable	Lower CI	Mean	Upper CI
Cooler	Proximity to escape	-0.03	-0.01	0.00
Cooler	Elevation	-0.02	0.00	0.02
Cooler	Proximity to road	-0.12	-0.09	-0.06
Cooler	Proximity to trail	0.47	0.49	0.52
Cooler	Solar radiation	-0.10	-0.08	-0.06
Cooler	Proximity to snow	-0.02	-0.01	0.01
Warmest	Proximity to escape	0.12	0.15	0.19
Warmest	Elevation	-0.45	-0.39	-0.32
Warmest	Proximity to road	-0.04	0.04	0.12
Warmest	Proximity to trail	0.30	0.36	0.42
Warmest	Solar radiation	-0.02	0.02	0.06
Warmest	Proximity to snow	-0.23	-0.19	-0.14
Difference	Proximity to escape	0.12	0.16	0.20
Difference	Elevation	-0.46	-0.39	-0.32
Difference	Proximity to road	0.04	0.13	0.21
Difference	Proximity to trail	-0.20	-0.13	-0.07
Difference	Solar radiation	0.06	0.10	0.15
Difference	Proximity to snow	-0.23	-0.18	-0.13
